# ASSIGN score and cancer risk in the Scottish Heart Health Extended Cohort (SHHEC) study

**DOI:** 10.1038/s44276-024-00102-5

**Published:** 2024-10-01

**Authors:** Catherine A. Fitton, Mark Woodward, Jill JF. Belch

**Affiliations:** 1 The Institute of Cardiovascular Research, Division of Molecular and Clinical Medicine, University of Dundee, Ninewells Hospital, Dundee, UK; 2 The George Institute for Global Health, School of Public Health, Imperial College London, London, UK; 3 The George Institute for Global Health, University of New South Wales, Sydney, NSW Australia

## Abstract

**Background:**

The aim of this work was to determine whether the ASSIGN cardiovascular disease (CVD) score, a 10-year CVD risk score used in primary care in Scotland, could additionally detect cancer risk.

**Methods:**

18,107 participants were recruited to the Scottish Heart Health Extended Cohort (SHHEC) study between 1982 and 1995. Information on health and lifestyle were collected, along with blood and urine, and participants were followed up via record linkage to 2017. Cox proportional hazards were used to estimate HRs (95% CIs) for time to cancer diagnosis.

**Results:**

A total of 5046 cases of cancer were reported during the follow up period. ASSIGN was significantly associated with a diagnosis of cancer, with a 2.3–3.4% increase in risk of cancer per 1-point increase of ASSIGN. The components of ASSIGN predominantly associated with the risk of cancer were age (HR 1.52; 95% CI 1.48–1.56, cholesterol level (HR 1.11; 95% CI 1.08–1.13), diabetes status (HR 1.24; 95% CI 1.01–1.53), and systolic blood pressure (HR 1.16; 95% CI 1.13–1.19).

**Conclusion:**

ASSIGN could be used not only to predict CVD, but also to predict cancer risk in patients. This needs to be validated in further cohorts.

## Background

In the UK, there are around 367,000 cancer cases reported a year [1]; however, population estimates do not reflect risk to the individual patient. Risk calculators have been developed for specific types of cancer, such as bowel and breast cancers [2, 3] and, while there are several well-known cancer risk factors, such as smoking, age and obesity, there is currently no way to quantify an individual’s risk of developing any cancer in their lifetime. Therefore, developing a risk scale for any cancer risk would be a useful, and potentially life altering, tool.

ASSIGN was developed in 2006 to provide a cardiovascular disease (CVD) risk scale in Scotland [4]. The ASSIGN data base came from the Scottish Heart Health Study [5], which recruited random samples of men and women aged 40–59 years across 25 districts of Scotland from 1984 to 1987. The Scottish MONICA Project recruited in Edinburgh and north Glasgow in 1986, north Glasgow again in 1989 and 1995, ages 25–64 and 1992, ages 25–74 [6]. Participants completed a questionnaire for a survey clinic where cardiovascular risk factors were measured and gave permission for follow-up through routine records. Participants qualified for analysis if they had risk-factor data, permitted follow up, were aged 30–74 years at recruitment and reported neither coronary heart disease nor stroke, and did not have preceding hospital discharge diagnoses of these, transient ischaemic attacks, or cancer. ASSIGN is easy to access and use [7], with applications for General Practitioners to facilitate discussions regarding a patient’s lifestyle and risk of disease. As many risk factors are common between CVD and cancer, it would be reasonable to hypothesise that a CVD risk scale could be also used for cancer risk The aim of this study was to investigate whether the ASSIGN score could be also used to predict cancer risk.

## Methods

### Cohort

We chose to evaluate ASSIGN as a risk score for cancer in the same study population used to develop ASSIGN as a cardiovascular risk score: the Scottish Heart Health Extended Cohort (SHHEC) study. SHHEC has been described in detail previously [8], but briefly, it combines the Scottish Heart Health Study (SHHS) [5, 9] and the Scottish MONICA study [6], where patients were randomly recruited across 23 districts of Scotland between 1984 and 1987, and from Edinburgh and North Glasgow 1986–1995 retrospectively. Both men and women, aged 40–59 y were recruited in SHHS and aged 25–75 y were recruited for MONICA, totalling 18,107 for SHHEC. Personal details and blood and urine samples were taken at recruitment, as described in detail [8]. Cohort variables were measured once, at recruitment. Permission was given for long term follow up via record linkage. Cohort characteristics have been described previously [8].

### Cancer data

Participants in SHHEC had their Community Health Index (CHI) numbers linked with the Scottish Morbidity Record 06 (SMR06), which registers cancer cases, and National Records of Scotland (NRS), which records deaths. The SMR06 records all cancer cases reported, including both malignant and benign neoplasms. We identified relevant cancer cases by using the following ICD10 codes: C00-97 for any cancer (excluding C43–44); C00–80 for solid cancers (excluding C43–44); C81–96 for haematolymphoid cancers. As there have been several versions of ICD codes used over the years, eDRIS convert all past ICD codes to the most recent version when providing the data to researchers, meaning only one version of ICD is required (ICD10 2016 version).

### ASSIGN formula

The ASSIGN score [4] was developed in 2006 to provide a tool to estimate cardiovascular disease risk. The development and the validity of the scale, as well as the full formula, has been previously published in full [4]. The ASSIGN score combines the following risk factors: age, total cholesterol, HDL cholesterol, systolic blood pressure, diabetes, family history of CVD, cigarettes per day and Scottish Index of Multiple Deprivation (SIMD) score and is calculated separately for women and men. SIMD is a socio-economic score used in Scotland, comprised of the following components: employment, income, health, crime, housing, education and access to common amenities [10]. The current SIMD score runs from 0 to 100, with the higher the value, the higher the socio-economic background.

To enable use of ASSIGN score for healthcare providers outside Scotland, we have also performed the analyses without SIMD.

### Statistical methods

Cox proportional hazard models were used to estimate hazard ratios with 95% confidence intervals (HR 95% CI), while allowing for different enrolment time of participants. Survival was counted to the first qualifying event (cancer diagnosis). No additional variables were added to the analysis.

ASSIGN factors that are continuously distributed factors were assessed as increase per standard deviation. Due to the large number of zero values for cigarettes per day, the data was split into 5 groups (with zero as the reference group). Diabetes, sex and family history of CVD were analysed as dichotomous factors, with “no” or male set as the reference level for analysis. The ASSIGN score was partitioned into fourths, using the 25th, 50th, and 75th centiles. The lowest fourth was set as the reference level for the analysis. Absolute cancer risk was calculated as a percentage, by dividing number of cancer cases by ASSIGN group total. This was calculated for both the original ASSIGN score, and the ASSIGN score without SIMD. Groups used were as follows: 0, 1–10, 11–20, 21–30, 31–40, 41–50, 51–60, 61–70, 71–80, 81–90, 90–99, 100.

### Ethics & permissions

Permission to evaluate this study was given by the electronic Data Research and Innovation Service (eDRIS)’s Public Benefit and Privacy Panel for Health and Social Care (PBPP number: 1516-0578/2223-0068). eDRIS is a service which provides a single point of contact for researchers to assist in data access via a secure analytical environment. eDRIS is designed to assist the investigator to uphold the Guiding Principles for Data Linkage and is a National Services Scotland (NSS) service. An agreement was signed between the University of Dundee/NHS Tayside and eDRIS using a NSS eDRIS User Agreement Reference 1516-0578/2223-0068. For the original clinical study ethical approval was received in 1984 from the Tayside Ethics Committee, approval no 39/84. All participants gave informed consent in writing at time of recruitment for their blood to be taken and data to be used for study purposes. Participants also gave permission for long term follow up via record linkage. The study was performed in accordance with the Declaration of Helsinki.

## Results

Of the 18,107 enrolled in SHHEC, by end of follow up in December 2017, 5046 had been diagnosed with a cancer. Cohort details for ASSIGN variables are given in Table 1, and cancer types are given in Supplementary Table 1. Within the cohort, cancer diagnosis was significantly associated with higher age, smoking more cigarettes per day, being male, higher SBP, lower SIMD score and a higher ASSIGN score (Table 1).

**Table 1:** Baseline mean (standard deviation) or count (%) data for ASSIGN variables by cancer outcome for the SHHEC cohort (*n* = 18,107)

Variable	No cancer (*N* = 12,360)	Cancer (*N* = 5747)	*P* value
Age (year)	47.8 (8.4)	51.7 (7.6)	<0.001
Cigs/day	7.5 (10.6)	8.4 (11.1)	<0.001
Sex (F)	6158 (52.0%)	3087 (49.2%)	<0.001
Total cholesterol (mg/L)	6.3 (1.2)	6.4 (1.2)	0.183
HDL cholesterol (mg/L)	1.5 (0.4)	1.5 (0.4)	0.256
Diabetes (Y)	232 (2.0%)	98 (1.6%)	0.053
Family history (Y)	3538 (29.9%)	1852 (29.5%)	0.938
SBP (mmHg)	131.4 (20.1)	133.4 (20.2)	<0.001
SIMD	28.0 (20.5)	27.2 (19.6)	0.041
ASSIGN	11.3 (10.3)	13.6 (10.8)	<0.001
ASSIGN (no SIMD)	20.1 (28.89)	21.9 (27.5)	<0.001

*mg/L* miligrams per litre, *mmHg* milligrams mercury, *CVD* cardiovascular disease, *SIMD* Scottish Index of Multiple Deprivation.

The ASSIGN score and the variables that contribute to the score, were significantly associated with the risk of developing cancer over the study period (Table 2). Age was the most significantly associated variable for any cancer, and when split into solid or haematolymphoid cancers (Table 2). SBP and being male were associated with all types of cancer assessed. Smoking, total cholesterol and a SIMD were significantly associated with a diagnosis of any cancer, or solid only, but not with haematolymphoid cancers. While diabetes and HDL cholesterol were associated with any cancer, but were not significantly associated with either solid or haematolymphoid cancers when assessed individually (Table 2).

**Table 2:** Hazard ratios for cancer, solid and haematolymphoid cancers associated with the individual ASSIGN factors.

Variable	Any cancer (*n* = 5747)		Solid only (*n* = 5489)		Haematolymphoid only (*n* = 352)	
	HR (95% CI)	*P* value	HR (95% CI)	*P* value	HR (95% CI)	*P* value
Age (years)	1.519 (1.483–1.556)	<0.001	1.524 (1.487–1.561)	<0.001	1.399 (1.268–1.543)	<0.001
Cigarettes/day	1.086 (1.071–1.102)	<0.001	1.088 (1.072–1.104)	<0.001	0.988 (0.929–1.052)	0.707
Total cholesterol (mg/L)	1.108 (1.082–1.134)	<0.001	1.115 (1.088–1.142)	<0.001	1.003 (0.909–1.108)	0.950
HDL (mg/L)	0.971 (0.948–0.996)	0.019	0.976 (0.952–1.001)	0.057	0.933 (0.842–1.034)	0.186
Sex (Ref: male)	0.937 (0.796–0.880)	<0.001	0.841 (0.799–0.885)	<0.001	0.683 (0.554–0.843)	<0.001
Diabetes (Ref: no)	1.244 (1.012–1.529)	0.038	1.224 (0.990–1.512)	0.062	1.187 (0.491–2.871)	0.703
SBP (mmHg)	1.158 (1.132–1.185)	<0.001	1.158 (1.131–1.185)	<0.001	1.183 (1.075–1.301)	<0.001
Family history of CVD	1.010 (0.956–1.067)	0.729	1.009 (0.954–1.067)	0.762	0.983 (0.781–1.237)	0.883
SIMD	1.074 (1.050–1.099)	<0.001	1.073 (1.049–1.099)	<0.001	1.060 (0.965–1.164)	0.227
ASSIGN	1.034 (1.031–1.036)	<0.001	1.034 (1.032–1.037)	<0.001	1.023 (1.012–1.034)	<0.001
ASSIGN (no SIMD)	1.003 (1.002–1.004)	<0.001	1.003 (1.002–1.004)	<0.001	1.002 (0.999–1.006)	0.195

Sex, diabetes and family history of CVD assessed as factors (yes vs no), cigarettes and others are assessed per standard deviation higher.

*mg/L* miligrams per litre, *mmHg* milligrams mercury, *CVD* cardiovascular disease, *SIMD* Scottish Index of Multiple Deprivation, *HR* Hazard Ratio, *CI* confidence interval.

The ASSIGN score was significantly associated with cancer diagnosis, per 1-point increase of ASSIGN (HR 1.034; 95% CI 1.031–1.036). A 1-point increase in the ASSIGN score was associated with a 2.3–3.4% increase in cancer risk (Table 2).

When assessed in fourths, the ASSIGN score was associated with a 24.3–33.0% increased risk of cancer (Table 3).

**Table 3:** Hazard ratios for cancer, solid and haematolymphoid cancers by fourths of the ASSIGN score, and per fourth higher.

Fourths	Any cancer		Solid cancer		Haematolymphoid	
	HR (95% CI)	*P* value	HR (95% CI)	*P* value	HR (95% CI)	*P* value
ASSIGN (original)						
1	Reference		Reference		Reference	
2	1.35 (1.23–1.49)	<0.0001	1.37 (1.25–1.51)	<0.0001	1.09 (0.77–1.56)	0.617
3	1.81 (1.66–1.98)	<0.0001	1.83 (1.67–2.01)	<0.0001	1.30 (0.91–1.85)	0.145
4	2.65 (2.42–2.90)	<0.0001	2.69 (2.45–2.95)	<0.0001	1.93 (1.36–2.73)	0.0002
Per fourth higher	1.38 (1.34–1.42)	<0.0001	1.39 (1.35–1.43)	<0.0001	1.24 (1.11–1.39)	0.0002
ASSIGN (no SIMD)						
1	Reference		Reference		Reference	
2	1.48 (1.36–1.61)	<0.0001	1.50 (1.37–1.64)	<0.0001	1.26 (0.91–1.74)	0.167
3	2.16 (1.99–2.35)	<0.0001	2.19 (2.01–2.38)	<0.0001	1.78 (1.30–2.43)	0.0003
4	2.34 (2.15–2.54)	<0.0001	2.38 (2.18–2.60)	<0.0001	1.57 (1.12–2.21)	0.009
Per fourth higher	1.33 (1.29–1.36)	<0.0001	1.33 (1.30–1.37)	<0.0001	1.19 (1.07–1.31)	0.001

Results for the original ASSIGN score, and ASSIGN without SIMD are given. Quartile ranges for ASSIGN are: Q1 < 4.88; Q2 4.88–8.99; Q2 9.00–16.06; Q4 > 16.06. Quartile ranges for ASSIGN without SIMD are: Q1 < 4.70 ; Q2 4.7–10.1; Q3 10.1–20.5; Q4 > 20.5.

*HR* Hazard Ratio, *CI* confidence interval.

**Fig. 1:**
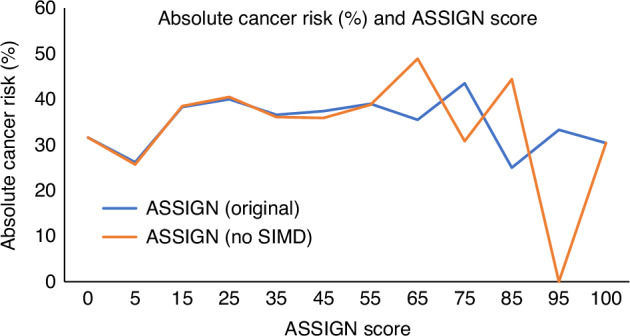
Absolute cancer risk (%) and ASSIGN score. Absolute risk for both the original ASSIGN score and the ASSIGN score without SIMD is included.

Absolute cancer risk was calculated for groups across the ASSIGN score (Fig. 1).

## Discussion

We investigated whether ASSIGN, a pre-existing tool used to quantify cardiovascular risk in an individual patient, could be used to also predict risk of cancer. ASSIGN was highly correlated with cancer risk, with a higher association to diagnosis of any cancer and solid cancer than haematolymphoid cancers. For every one-point increase in ASSIGN score, there was a 2.3–3.4% increase in cancer risk.

Most cancer ‘risk scores’ target individual cancer types such as bowel [11], and breast [12]. ASSIGN assesses ‘general’ cancer risk, and therefore acts as an ‘early warning’ sign to the clinician, allowing deeper questioning to be undertaken about possible cancer symptoms. It also affords the clinician the opportunity to alert the patient to their higher cancer risk and to effect the lifestyle changes to risk factors such as smoking and exercise that the patient needs to achieve. There were not sufficient individual cases of different cancer types to assess whether ASSIGN better predicted any specific cancer type.

ASSIGN is an online tool that can be accessed freely to provide a quick and easy measure of risk of developing the western world’s two biggest killers and allow further discussion within primary care between clinical staff and the patient about their personalised risk score and their modifiable risk factors. Other cancer risk online tools for specific cancer types are available, such as the CanRisk tool, which predicts breast and ovarian cancer [13].

Modelling cancer risk using national electronic health databases to produce models to predict cancer risk in populations has already been demonstrated [14]. Advancement of technology and techniques could enable these, and ASSIGN, to be further developed by machine learning and artificial intelligence in the future [15, 16]. Molecular biomarkers are also likely to come to the fore [17] and we are already seeing this in the literature [18, 19], but until these become fully validated and available, this ASSIGN score can alert the clinician, not only to their patient’s risk of CVD but also of cancer.

Determining whether a patient is at risk of developing cancer is an important first step to providing advice and preventative treatment. Cancer risk can be reduced by modifying certain lifestyle choices, such as stopping smoking, and losing weight [20]. Furthermore, there may be preventative treatments that can be used for both the prevention of cancer and cardiovascular disease. The use of low dose aspirin has widely been reported to decrease risk of CVD events, however it is not currently recommended for preventative treatment, due to the risk of bleeding in older patients [21]. Recent evidence has shown that daily aspirin may also reduce the risk of developing cancer [22]; this along with the wide availability and relatively low cost of aspirin makes it a particularly useful preventative in those who are younger, but with a high ASSIGN score.

### Strengths

ASSIGN has already been developed, requires no modification for those in Scotland, and is free to access by the general public as well as by Medical Practitioners. It is a robust risk predictor for an individual’s risk for both CVD and cancer.

### Limitations

ASSIGN doesn’t include some common cancer risk factors, such as weight and family history of cancer. While these may improve the accuracy and specificity of the score, this was an investigation to determine whether a previously developed ‘in use’ CVD score could be used as a proxy for cancer risk, without any modification.

ASSIGN may provide a useful tool to quantify cancer risk at the patient level in Scotland without the need to design a specific cancer scale. Using ASSIGN for both CVD and cancer risk can help inform both the GP and the patient regarding the individual level of risk and about modifiable risk factors that may reduce their risk of disease. This scale could both be developed to include cancer specific risk factors such as family history of cancer, and weight whilst being validated in other cohorts.

## Supplementary information

Supplemental table 1

## Data Availability

Data for this study have been stored in the safe haven, eDRIS. Data access is thus via a secure analytical environment. eDRIS is a National Services Scotland (NSS) service. An agreement was signed between the University of Dundee/NHS Tayside and eDRIS using a NSS eDRIS User Agreement.

## Code availability

The code used in this project can be provided by reasonable request to the authors. The data that support the findings of this study are owned and maintained by eDRIS. Data are available following a successful PBPP application to eDRIS, following their guidelines. To apply to eDRIS to access for data, contact phs.edris@phs.scot.
