# Supplementary material for: ASSIGN score and cancer risk in the Scottish Heart Health Extended Cohort (SHHEC) study
Source: BJC Rep. 2024 Oct 1;2:75. doi: 10.1038/s44276-024-00102-5 (PMC11523964; doi:10.1038/s44276-024-00102-5)
Supplement: Supplementary file 1 — Supplemental table 1 [file 44276_2024_102_MOESM1_ESM.docx]

Supplemental table 1. Types of cancer reported during the study period. Cancer cases include cases after first diagnosis. *not included in total due to sensitive data.

| ICD10 code | Site of malignant neoplasm | n |
| --- | --- | --- |
| C00-14 | Lip, oral cavity and pharynx | 122 |
| C15-26 | Digestive organs | 1264 |
| C30-39 | Respiratory and intrathoracic organs | 1094 |
| C40-41 | Bone and articular cartilage | ≤5* |
| C45-49 | Mesothelial and soft tissue | 65 |
| C50 | Breast | 696 |
| C51-58 | Female genital organs | 291 |
| C60-63 | Male genital organs | 543 |
| C64-68 | Urinary tract | 335 |
| C69-72 | Eye, brain and other parts of central nervous system | 64 |
| C73-75 | Thyroid and other endocrine glands | 24 |
| C76-80 | Ill-defined, secondary and unspecified sites | 183 |
| C81-96 | Malignant neoplasms, stated or presumed to be primary, of lymphoid, haematopoietic and related tissue | 356 |
